# Carotenoid Production from Microalgae: Biosynthesis, Salinity Responses and Novel Biotechnologies

**DOI:** 10.3390/md19120713

**Published:** 2021-12-20

**Authors:** Yuanyuan Ren, Han Sun, Jinquan Deng, Junchao Huang, Feng Chen

**Affiliations:** 1 Institute for Food and Bioresource Engineering, College of Engineering, Peking University, Beijing 100871, China; 1701111648@pku.edu.cn; 2 Shenzhen Key Laboratory of Marine Microbiome Engineering, Institute for Advanced Study, Shenzhen University, Shenzhen 518060, China; sunhanias@163.com (H.S.); 1900392002@email.szu.edu.cn (J.D.); 3 Institute for Innovative Development of Food Industry, Shenzhen University, Shenzhen 518060, China

**Keywords:** carotenoids, microalgae, salt stress, seawater cultivation, Internet of Things

## Abstract

Microalgae are excellent biological factories for high-value products and contain biofunctional carotenoids. Carotenoids are a group of natural pigments with high value in social production and human health. They have been widely used in food additives, pharmaceutics and cosmetics. Astaxanthin, β-carotene and lutein are currently the three carotenoids with the largest market share. Meanwhile, other less studied pigments, such as fucoxanthin and zeaxanthin, also exist in microalgae and have great biofunctional potentials. Since carotenoid accumulation is related to environments and cultivation of microalgae in seawater is a difficult biotechnological problem, the contributions of salt stress on carotenoid accumulation in microalgae need to be revealed for large-scale production. This review comprehensively summarizes the carotenoid biosynthesis and salinity responses of microalgae. Applications of salt stress to induce carotenoid accumulation, potentials of the Internet of Things in microalgae cultivation and future aspects for seawater cultivation are also discussed. As the global market share of carotenoids is still ascending, large-scale, economical and intelligent biotechnologies for carotenoid production play vital roles in the future microalgal economy.

## 1. Introduction

Carotenoids are a class of terpenoid pigments with C40 backbones and are health-promoting for human daily diets. Up until now, over 1100 carotenoids have been discovered, and they exist in various species [1], especially in aquatic creatures, microorganisms and terrestrial plants. Carotenoids are very common in our daily lives; tomato fruit is rich in lycopene (pink red), maize corn is abundant with zeaxanthin (yellow) and carrots and *Dunaliella salina* are well known for producing β-carotene (orange) [1]. Carotenoids ingested by humans can work as precursors of Vitamin A [2], reduce free radicals [2] and repair damaged retina [3]. Moreover, they can also reduce the risks of some diseases as they have anti-cancer, anti-inflammatory and anti-obesity properties [4]. Hence, carotenoids have been widely used in the food, feed, cosmetic and pharmaceutical industries.

With the development of modern biotechnologies and market concern for food safety, the demand for carotenoids from natural sources is increasing remarkably. Compared with plant-derived carotenoids, those from microorganisms are more efficient, have lower cost and are not limited by regions and seasons. However, the carotenoids market is still mainly occupied by chemically synthetic products (80–90%), with a much lower portion of natural sources (10–20%) [5]. Thus, choosing low-cost, profitable and safe microorganisms for carotenoid production is drawing extensive interest.

Microalgae are diverse and abundant. They can adapt to different cultivation conditions, grow rapidly and accumulate a high amount of desirable bioproducts, such as fatty acids, proteins and carotenoids. The global algae market is expected to reach 970 million U.S. dollars by the end of 2025 [6] and that of carotenoids is expected to reach 2 billion U.S. dollars by 2026, mainly including food and beverages (26.1%), pharmaceuticals (9.2%), cosmetics (6.5%) and dietary supplements (23.5%) [7]. Some species, such as *Chlorella* and *Spirulina*, have GRAS status and are well accepted as health foods [8].

Microalgae have versatile metabolic modes and tend to accumulate different metabolites. Different trophic modes (autotrophy, mixotrophy, heterotrophy), culture conditions (light, pH, dissolved oxygen) and nutrient availability (repletion or depletion) will lead carbon skeletons to have significantly different cell compositions. For primary metabolites, light and adequate nutrients are essential for active growth. On the contrary, adverse stresses, such as nutrient deficiency, high light and salt stress, are key factors that arouse the defense mechanisms of microalgae and accumulate secondary metabolites to survive.

Salt stress is a common stress factor in natural environments, especially for freshwater microalgae. Salt stress can lead to oxidative damage, chlorophyll degradation and inhibit photosynthesis and growth [9]. To adapt to salt stress, microalgae have evolved a survival strategy to guarantee the balance of growth and stress responses. During the adaptation to stress, some microalgae may become cysts and accumulate secondary metabolites, such as astaxanthin and triacylglycerol [10]. However, serious salt stress can be fatal to microalgae when the cells are in low concentration or poor viability. Thus, two-stage cultivation, balancing cell density and metabolites accumulation, is a popular strategy in the practical production of high-value compounds from microalgae [11]. Due to the shortage of freshwater resources, overcoming the salt tolerance problem of freshwater microalgae is also of invaluable significance for microalgae cultivation in seawater.

This review comprehensively introduces the production of high-value carotenoids and their beneficial effects on human health. The latest research results are summarized, and biotechnology topics are focused on, such as yields of microalgae-derived carotenoids and microalgal cultivation strategies. Meanwhile, the contribution of salt stress on the growth and carotenoid accumulation of microalgae is also discussed. Moreover, considering modern advanced technologies, the application prospects of the Internet of Things in innovative biotechnology are discussed. In summary, this article aims to provide readers with comprehensive knowledge of microalgae-derived carotenoids and their related biotechnologies, coupled with salt-stress treatment or endurance.

## 2. Health-Promoting Carotenoids from Microalgae and Their Biofunctions

In the global market for carotenoids, β-carotene will reach a value of 620 million U.S. dollars by the end of 2026, lutein will reach 357.7 million U.S. dollars by the end of 2024 and astaxanthin will surpass 800 million in 2026 [1]. In addition, other health-promoting carotenoids, such as fucoxanthin [12] and zeaxanthin [13], are also drawing customers’ attention. 

Microalgal biomass can be harvested regardless of seasons and districts. Other biofunctional metabolites in microalgae can also be processed as value-added products to increase revenue. Carotenoid production from microalgae requires less labor and an easy harvesting process. Compared to terrestrial plants, microalgae have higher photosynthetic efficiency and a superior growth rate. Hyperaccumulating strain selection and the culture condition optimization required to achieve high biomass and maximum carotenoid productivities are the bottlenecks to be resolved at present. In this section, we comprehensively describe carotenoid biosynthesis in microalgae and list several microalgae-derived carotenoids and their biofunctions.

### 2.1. Carotenoid Biosynthesis in Microalgae

Carotenoids in microalgae can be categorized as the primary ones related to photosynthesis, and the secondary ones accumulated under adversity [14]. In this review, we mainly focus on functional microalgal carotenoids, including lutein, β-carotene, astaxanthin, zeaxanthin and fucoxanthin.

**Figure 1:**
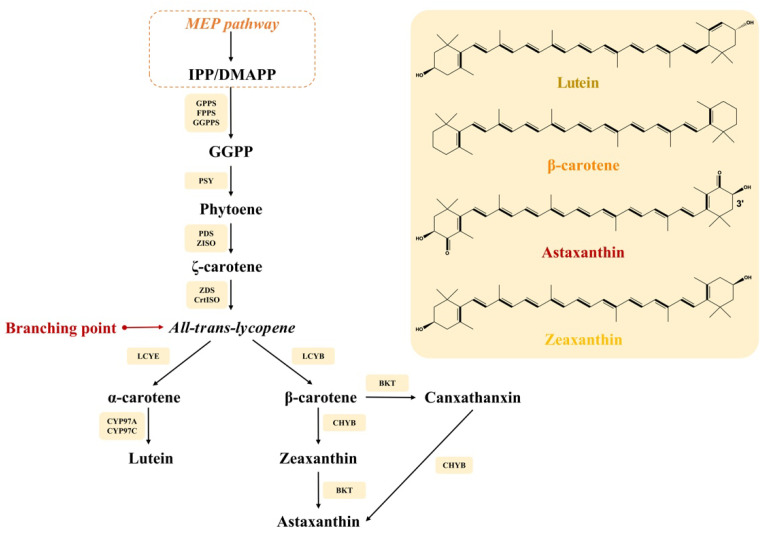
Biosynthesis pathways of carotenoids in microalgae and their chemical structures. MEP: methylerythritol phosphate; IPP: isopentenyl pyrophosphate; DMAPP: dimethylallyl diphosphate; GPPS: geranyl diphosphate synthase; GGPPS: geranylgeranyl pyrophosphate synthase; GGPP: geranylgeranyl pyrophosphate; PSY: phytoene synthase; Z-ISO: ζ-carotene isomerase; ZDS: ζ-carotene desaturase; CrtISO: carotene isomerase; LCYE: lycopene epsilon cyclase; CYP97A: cytochrome P450 beta hydroxylase; CYP97C: cytochrome P450 epsilon hydroxylase; LCYB: lycopene β-cyclase; BKT: β-carotene ketolase; CHYB: β-carotene hydroxylase.

The biosynthetic pathways of carotenoids involve several intermediates and key enzymes (shown in Figure 1) [15,16]. First, through the methylerythritol phosphate pathway (MEP) in the chloroplast, isopentenyl pyrophosphate (IPP, C5) and its isomer dimethylallyl diphosphate (DMAPP, C5) are catalyzed at a ratio of 3:1 by geranyl diphosphate synthase (GPPS) and geranylgeranyl pyrophosphate synthase (GGPPS) to geranylgeranyl pyrophosphate (GGPP, C20). In *H. pluvialis*, this step from C5 to C20 can be catalyzed by GGPPS only [10]. Phytoene synthase (PSY) condenses two molecules of GGPPS into (15*Z*)-phytoene (C40), which is desaturated and isomerized into lycopene by phytoene desaturases (PDS), ζ-carotene isomerase (Z-ISO), ζ-carotene desaturase (ZDS) and carotene isomerase (CrtISO).

Lycopene may flow to two branches, α-branch and β-branch. Lycopene ε-cyclase (LCYE) and lycopene β-cyclase (LCYB) catalyze lycopene to α-carotene, and then cytochrome P450 beta hydroxylase (CYP97A) and cytochrome P450 epsilon hydroxylase (CYP97C) convert α-carotene to lutein. As for the β-branch, lycopene is converted by LCYB to β-carotene. For the microalgae capable of astaxanthin accumulation, such as *Haematococcus pluvialis* and *Chromochloris zofingiensis*, β-carotene may also flow to different branches. β-carotene can first be catalyzed by β-carotene ketolase (BKT) to canthaxanthin and then to astaxanthin by β-carotene hydroxylase (CHYB). β-carotene can also flow to zeaxanthin first by CHYB and then to astaxanthin by BKT [15]. This also reflects the diversity of microalgae metabolism. Up until now, the exact biosynthesis pathway of fucoxanthin is still unclear.

### 2.2. Health-Promoting Carotenoids and Their Production from Microalgae

#### 2.2.1. Lutein

Lutein, (*3R, 3′R, 6′R*)-β, ε-carotene-3, 3′-diol, is a natural antioxidant and has drawn interest for its health-promoting functions. Human metabolism cannot synthesize lutein, and lutein uptake in a suggested dose (6 mg day^−1^) has been proved to be beneficial for human health. Lutein has potentials in free radical scavenging for skin health and can also prevent age-related macular degeneration (AMD) and Alzheimer’s Disease (AD) [3,17]. Absorbed lutein can accumulate in human retina, filter blue light and, thus, protect eyesight. Biofunctional carotenoids and their natural sources, biofunctions and recommended doses are listed in Table 1.

Orange-yellow fruits like mango, broccoli and other green leafy vegetables are dietary sources of lutein [22].The marigold flower is the main source of natural lutein and lutein esters. Lutein contents in different species of marigold petals range from 17 to 570 mg/100 g [23]. However, there are some drawbacks of this source, such as mandatory harvesting in specific seasons and time-consuming petal separation. Other sources containing lutein also have such disadvantages as low concentration (corn residues, leafy green vegetables) and low bioavailability (egg yolk, crustaceans) [24].

The production of lutein from microalgae may avoid these troubles. Microalgae can accumulate considerable biomass concentrations and accumulate lutein under suitable culture conditions. As a primary xanthophyll carotenoid, lutein is an antenna pigment in the light-harvesting complex (LHC) of microalgal photosynthetic apparatus. When light intensity is too high, lutein can reduce oxidative damage by non-photochemical quenching (NPQ) [25]. Thus, illumination is a key environmental factor for lutein accumulation.

Compared with marigold-originated lutein, lutein in microalgae exists in free form. The feasibility and economic competitiveness of using microalgae to replace the marigold flower to produce lutein has been widely discussed [23,24], including harvesting cost, extraction and processing methods and bioavailability analysis. Up until now, many microalgae subgenera have been investigated for lutein production, such as *Scenedesmus*, *Chlorella*, *Coccomyxa*, *Parachlorella* and *Tetraselmis* [25].

To achieve both high content and productivity of carotenoid from microalgae, various cultivation factors are globally considered, including trophic mode, carbon source, fed-batch, semi-continuous operation, light intensities and light/dark cycles, etc. [25]. Mixotrophic is popular in lutein production from various microalgae capable of glucose uptake, such as *Chlorella minutissima* [26], *Chlorella sorokiniana* MB-1-M12 [27] and *Chlorella* sp. GY-H4 [28]. The lutein production of *Chlorella* sp. GY-H4 under 20 g/L glucose (10.5 mg/L/day, 63 mg/L) was higher than that under 10 g/L glucose (7.3 mg/L/day, 44 mg/L) [28]. By adding NaAc, *Chlorella sorokiniana* MB-1-M12 can accumulate 7.39 mg/g DW of lutein [27]. Two-stage cultivation, such as the mixotrophic-photoautotrophic process of *Chlorella sorokiniana* FZU60, also achieved an efficient production at 8.25 mg/L/day [29]. Moreover, food waste [28], poultry litter waste [30] and wastewater [31] have been applied to microalgae cultivation for lower cost. A recently isolated microalga, *Parachlorella* *sp.* JD-076, is high in lutein productivity at 25 mg/L/day via regulation of illumination and CO_2_ supply [32]. Results of satisfactory lutein production from microalgae are summarized in Table 2 with details of contents and productivities.

#### 2.2.2. Astaxanthin

Astaxanthin (3, 3′-dihydroxy-β, β’-carotene-4, 4′-dione) is a natural pigment existing in various species of aquacultures [18]. It is acknowledged as the strongest natural antioxidant, which is 65 times that of vitamin C and 10 times higher than other carotenoids [51]. Astaxanthin has been widely applied in functional foods, pharmaceuticals and cosmetics, with its outstanding potential in free radical scavenging and its anti-aging, anti-inflammatory, anti-hypertensive and anti-cancer properties [10].

Currently, the astaxanthin market is mostly occupied by chemically synthesized products. It is a mixture of three stereoisomers, namely (*3R,3′R*), (*3R, 3′S*) and (*3S, 3′S*) astaxanthin at the ratio of 1:2:1 [52], which is structurally heterogeneous and inefficient for biological uptake. For now, microalgae-derived astaxanthin only occupies 1% of the current astaxanthin market. Additionally, the production cost of natural astaxanthin (USD 1800/kg) is much higher than that of the chemically synthesized one (USD 1000/kg) [53]. Thus, improving the productivity of astaxanthin by microalgae is essential for commercialization.

Microalgae-derived astaxanthin has been successfully approved by the Food and Drug Administration (FDA) for direct human consumption [8]. In microalgae, astaxanthin is a secondary carotenoid accumulated under adversity. It can mediate cellular redox imbalance for microalgae to survive oxidative stress [14]. As for the current main source of natural astaxanthin, *Haematococcus pluvialis* can accumulate up to 5% DW of astaxanthin with attached cultivation [37] and has been used for biotechnology industry for astaxanthin production with an annual yield of 300 tons of biomass [54]. Under adverse growth conditions, *H. pluvialis* transforms from green and motile cells to red cysts cells with high astaxanthin content [52]. Many pieces of research have exploited strategies for boosting astaxanthin accumulation, such as nutrient deficiency, high light, adding extra chemicals and some combined strategies [10].

*Chromochloris zofingiensis* is another promising candidate for astaxanthin production. In comparison to *H. pluvialis*, *C. zofingiensis* can take up glucose as a carbon source and achieve high biomass concentration in mixotrophy [55]. It has drawn a lot attention for its broad capability for producing carotenoids, lipids and exopolysaccharides under different conditions [56]. Astaxanthin content in *C.* *zofingiensis* can reach 6.5 mg/g DW [38], and robust biotechnological traits of *C. zofingiensis* may be competitive as a better organism for large-scale astaxanthin production. Results of satisfactory astaxanthin production from microalgae are summarized in Table 2 with details of contents and productivities.

#### 2.2.3. β-Carotene

β-carotene (C_40_H_56_) is a pure hydrocarbon carotenoid and commonly exists in plants (pumpkins, mango, carrots, etc.), fungi (*Phaffia rhodozyma*) and algae [57]. It is lipid-soluble and serves as the precursor of Vitamin A in human body, benefiting the treatment of night blindness [2]. Currently, β-carotene is widely used in foods and medicines for its excellent antioxidant, anti-cardiovascular and immune enhancement properties [57]. Although chemically synthetic β-carotene is low in cost, natural β-carotene is more acceptable to consumers.

β-carotene is a primary carotenoid in microalgae, responsible for transferring light energy to chlorophylls to expand the light absorbing spectrum [40]. Moreover, it is also the first ever commercialized high-value product from microalgae. β-carotene from microalgae has been approved by the FDA for direct human consumption [8].

The marine microalga *D. salina* can accumulate β-carotene up to 13% DW [58] when induced by different stress factors, such as high light, high temperature, nutrient deprivation and high salinity [59], which is the main source of natural β-carotene. As *D. salina* is capable of growing in high salinity, cultivation of *D. salina* in seawater with high yield of β-carotene is profitable, which may also avoid microorganism contamination. In addition, many species of microalgae can synthesize β-carotene. *Chlorella zofingiensis*, *Spirulina platensis* and *Caulerpa taxifolia* accumulate β-carotene at an average yield of 0.1–2% DW [40]. Studies have shown that the β-carotene content of *Spirulina* is 10 times that in carrots [57]. A mutant *C. zofingiensis bkt1* can accumulate high amounts of three carotenoids, as lutein (13.81 mg/g DW), β-carotene (7.18 mg/g DW) and zeaxanthin (7.00 mg/g DW) under high light and salt stress [33]. Results of satisfactory β-carotene production from microalgae are summarized in Table 2 with details of contents and productivities.

Moreover, the profiles of chemically synthesized and microalgae-derived β-carotene are different. The synthetic β-carotene only has (*all-E*)-isomer. In contrast, β-carotene from *D. salina* mainly contains three isomers, (*all-E*)-β-carotene (~42%), (9*Z*)-β-carotene (~41%), (15*Z*)-β-carotene (~10%) and others (~6%) [60]. As the (9*Z*)-β-carotene plays a key role in antioxidation, microalgae have potentials in producing low-cost and safer natural β-carotene for stronger biofunctions.

#### 2.2.4. Zeaxanthin

Zeaxanthin is a xanthophyll pigment existing in human eyes and skin. Together with lutein, zeaxanthin accumulates in the cornea as a macular pigment, protecting the retinal membrane from blue light and improving visual acuity [24]. Zeaxanthin also has potentials as an antioxidant, anti-inflammatory and for preventing neurological disease [20]. Maize, orange peppers and cooked scallions are good choices for dietary intake, while their contents are relatively as low at 16.3, 16.7 and 24.9 μg/g, respectively [13,22].

Currently, the marigold flower is applied for commercial production of zeaxanthin. Zeaxanthin content in different species of marigold petals ranges from 10 to 300 μg/g [61]. As the global number of people suffering from AMD [13] increases, the demand for natural sources with higher productivity of zeaxanthin is surging. The marine green alga *Chlorella ellipsoidea* produces zeaxanthin (4.26 mg/g DW) nine-fold over that produced by red pepper [44]. Additionally, microalgae-derived zeaxanthin partly exists in free form, rather than as mono-/di- esters in flowers and fruits of plants. Thus, microalgae are more valuable sources for processing zeaxanthin with higher productivity and bioavailability.

The production of zeaxanthin from microalgae is attractive and varies depending on microalgae species and cultivation factors. The favorable light condition for zeaxanthin varies in microalgae species. The zeaxanthin content of *C. zofingiensis bkt1* (5.69 ± 0.45 mg/g DW) was strongly enhanced by high light as compared with that of low light control (0.34 ± 0.04 mg/g DW) [33]. Under high light, combined with N-starvation, C. *zofingiensis bkt1* can accumulate 7.00 ± 0.82 mg/g DW. However, high light is not always preferred for microalgae to accumulate zeaxanthin. A mutant *D. salinazea1* can accumulate 5.9 mg/g DW of zeaxanthin under continuous low light (100 μmol photons m^−2^ s^−1^), higher than that transferred to high light (4.18 mg/g DW) [43]. As *D. salina* grows well in hypersaline medium, cultivation of this strain with seawater is potentially profitable. Results of satisfactory zeaxanthin production from microalgae are summarized in Table 2 with details of contents and productivities.

Additionally, downstream processing of zeaxanthin needs more attention, such as extraction and separation. Solvent systems have different efficiencies in zeaxanthin extraction from microalgae, and the maximum extraction of zeaxanthin (30.2 mg/g DW) was in chloroform: methanol (1:2) [41]. Pressurized liquid extraction was also applied to extract zeaxanthin from *C. ellipsoidea* [41]. More importantly, zeaxanthin shares similar chemical structures to lutein. Huang et al. developed an efficient method for the separation of zeaxanthin and lutein by ultra-high performance liquid chromatography (UHPLC) equipped with a Waters YMC Carotenoid C30 column [33].

#### 2.2.5. Fucoxanthin

**Figure 2:**
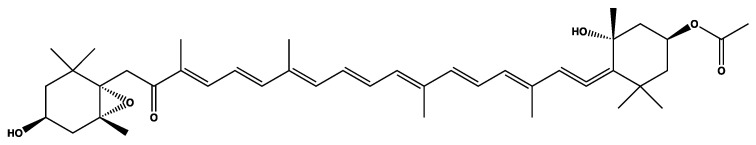
The chemical structure of (*all-E*)-fucoxanthin.

Fucoxanthin is a natural carotenoid that occupies nearly 10% of total natural carotenoid production [62]. The global production of fucoxanthin was about 500 tons in 2016 and was estimated to increase further with an annual rate of 5.3% [63]. As shown in Figure 2, it has a unique molecular structure with an allenic bond, a conjugated carbonyl, a 5,6-monoepoxide and acetyl groups. Fucoxanthin and its derivatives have shown potential anti-cancer [64], anti-inflammatory [65] and anti-obesity effects [46].

Currently, the main natural sources of fucoxanthin rely on some macroalgae species, such as *Laminaria japonica* and *Undaria pinnatifid*, which are common in Asian diets for iodine supplement [66]. However, the fucoxanthin contents in these macroalgae are relatively low (0.02–0.58 mg/g fresh weight) and not feasible for commercialization. Microalgae grow faster than macroalgae with higher productivity of carotenoids. Fucoxanthin is a primary light-harvesting carotenoid that transfers energy to LHC with high efficiency (>80%) [67] and may also protect microalgae from high light with its internal antioxidant property [47]. Up until now, research about producing fucoxanthin from microalgae is still relatively lacking.

Microalgae are potential candidates for fucoxanthin production. Fucoxanthin mainly exists in heterokont and haptophyte groups of algae (>20,000 species, especially in Synurophyceae (up to 26.6 mg/g DW), diatoms (up to 21.67 mg/g DW) and Prymnesiophyceae (up to 18.23 mg/g DW) [45]. Under low light intensity, carbon skeletons are diverted into carotenoids, especially fucoxanthin in *Isochrysis zhanjiangensis*, which can produce 23.29 mg/g DW of fucoxanthin [46]. The marine diatom *Odontella aurita* can accumulate 18.47 mg/g DW of fucoxanthin in a nitrogen-replete medium under low light [47]. Moreover, the purified fucoxanthin from *O. aurita* is (*all-E*)-fucoxanthin, which owns strong antioxidant ability and bioavailability. Maria et al. isolated a novel strain *Mallomonas sp.* (Synurophyceae) with the highest known content of fucoxanthin at 26.6 mg/g DW [45]. The highest ever reported fucoxanthin productivity was found in *Tisochrysis lutea* with 9.81 mg/L/day under batch culture with continuous chemostat dilution [49].

Diatom *Phaeodactylum tricornutum* also has a high fucoxanthin content at 16.33 mg/g DW [50]. As *P. tricornutum* is a model system for investigations and its genome has been sequenced, further exploitations of key genes related to fucoxanthin, fucoxanthin hyper-accumulating mutants and genetic modifications of diatoms for fucoxanthin production are expected in the near future. Results of satisfactory fucoxanthin production from microalgae are summarized in Table 2 with details of contents and productivities.

## 3. Salt-Stress Treatment for Carotenoid Production from Microalgae

Microalgae have flexible metabolic systems, which means that external environmental changes will affect biochemical components of microalgal cells. Stress factors, such as high light intensity, salinity, nitrogen starvation and high/low temperatures, will provoke metabolic changes in microalgae and lead to different cell compositions.

Though carotenoid production could be boosted by several environmental abiotic stress factors, salt-stress treatment is much more attractive because it is cheap, easy to operate and has great commercialization value for seawater culture of microalgae. Here, we elucidate the contributions of salinity in microalgal culture and production of carotenoids and summarize the current strategies of salt-stress treatment in carotenoid production.

### 3.1. Microalgal Responses to Salt Stress

Salt stress is one of the major abiotic stresses decreasing productivity of agriculture all over the earth [68,69]. Salinity inhibits plant growth, development, seed germination and yields. In contrast, plants have developed strategies to adapt to high salt concentrations, such as regulating stress hormones and growth hormones to balance growth and stress responses [69]. Currently, research on salt stress is more in-depth and comprehensive in higher plants, but there are a few studies on microalgae.

**Figure 3:**
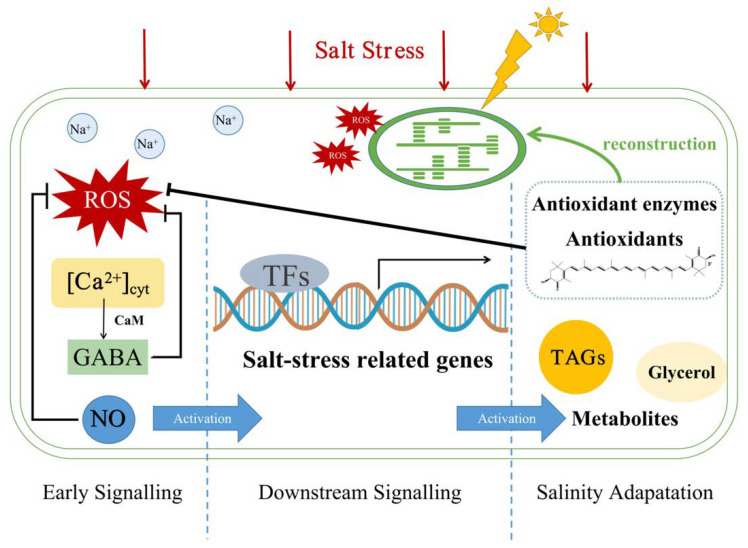
Possible mechanisms of responses of microalgae induced by salt stress.

Due to the fast growth rate of microalgae and their richness of high-value products, large-scale microalgae cultivation is imperative at present [6]. Microalgae are widely distributed in the ocean and freshwater, while salt stress is still a threat to the large-scale production of freshwater microalgae. As freshwater resources are becoming scarce today, the cultivation of freshwater microalgae with seawater is of great significance. On the other hand, appropriate salt stress shows promoting effects on secondary metabolite accumulation (such as secondary carotenoids and lipids) [70]. Therefore, it is of great significance to study the response of microalgae to salt stress. The possible responses in microalgae cells caused by salt stress are summarized in Figure 3.

Salt stress can affect microalgae on multiple levels, as physiological characteristics, gene expression and metabolic pathways. Salt stress can reduce cell growth, decrease chlorophyll content, inhibit photosynthesis and cause morphological changes in microalgae [9]. Under salt stress, the cell wall of *H. pluvialis* thickens, the cell volume becomes larger and gradually becomes immobile cysts [10]. These morphological changes are related to a series of in-depth signaling and downstream changes in both genetic and metabolic aspects.

#### 3.1.1. Early Signaling

Ca^2+^ is considered as a universal second messenger for the primary stress signals [71], acting in real-time in response to the imbalance of cell ion homeostasis under NaCl stress in plants [72]. Similar to plants, salt stress can elicit a temporary increase in cytosolic Ca^2+^ concentration ([Ca^2+^]_cyt_), which can regulate the activity of downstream effector proteins, such as calmodulin (CaM), Ca^2+^-dependent protein kinases and CaM-dependent protein kinases [73]. Moreover, the decarboxylation of glutamate to γ-aminobutyric acid (GABA) was regulated by the Ca^2+^/CaM protein.

GABA is a recently identified endogenous signaling molecule in plants, participating in cell growth and enhancing abiotic stress tolerance [74]. It is a non-protein amino acid derived from the decarboxylation of glutamate, and exogenous GABA can enhance plant resistance to abiotic stress by activating the GABA bypass pathways and TCA cycle. Under salt stress, GABA helps to maintain C/N balance and even acts as a scavenger of toxic ROS [75]. It is also related to nitric oxide (NO) accumulation under stress conditions as it amplifies NO stress signaling [74].

NO is an important molecule involved in plant growth, development and tolerance to abiotic stress. It plays important roles in resistance to drought, temperature (high or low), UV-B and heavy metal stress [76]. NO can also act as a signal in activating antioxidant enzyme defense against oxidative stress induced by salt stress [76]. By applying NO donors, such as sodium nitroprusside, plants under stress conditions showed restoration of chlorophyll to recover their damaged photosynthetic system [77].

Reactive oxygen species (ROS) are also second messengers induced by salinity stress, which is also associated with Ca^2+^ signaling [73]. ROS is a stress indicator in response to abiotic stress of microalgae, which can regulate cell growth and metabolites synthesis. Toxic ROS will lead to lipid peroxidation, membrane deterioration, DNA and protein damage [68]. To eliminate excessive ROS, antioxidant enzymes (such as superoxide dismutase and catalase) and antioxidants (such as carotenoids) in microalgae are two essential mechanisms evolved by organisms [78].

#### 3.1.2. Downstream Signaling

Salinity-induced signals may then have effects on the gene expression of microalgae cells, which is related to salt concentrations [79]. Low salt concentration can promote the growth of some microalgae, such as cultures of *Scenedesmus sp.* [80], *Botryococcus braunii* [81] and *H. pluvialis* [79]. Treatment of *H. pluvialis* with low-dose NaCl (12.5 mg/L) showed a promoting effect on biomass concentration (28% higher) and productivity (from 0.15 d^−1^–0.22 d^−1^). These effects were related to upregulation of growth-related genes, such as *rbcL*, *rbcS* and nitrate reductase gene (*NR*).

Under high-dose salt stress, both ionic and osmotic homeostasis need to be maintained. As for the halotolerant microalga *D. salina*, osmoregulation under osmotic stresses can be divided into two mechanisms. One is to maintain intracellular Na^+^ and K^+^ concentrations by the plasma membrane electron transport (redox) system, as Na^+^-ATPase and K^+^ carriers, and the other is to regulate glycerol concentrations inside to maintain the water potential inside and outside of cells [82]. Accumulation of glycerol as a soluble substance is a strategy for microalgae to keep osmotic homeostasis under salt stress, which has been demonstrated in *C. reinhardtii* [83] and *C. zofingiensis* [15]. Salt stress can also upregulate the genes related to starch catabolism (like pyruvate kinase, PK) and downregulate the genes for gluconeogenesis (like phosphoenolpyruvate carboxykinase, PEPCK), providing more building blocks for storage lipids (fatty acids and triacylglycerol, TAGs) and carotenoids in *C. zofingiensis* [15]. Salt-induced acetyl-coenzyme A carboxylase (ACCase) expression for fatty acid synthesis was also demonstrated in *Chlamydomonas* sp. [84], *Chlorella* sp. [85] and *Nitzschia* sp. [86].

Omics approaches like genomic, transcriptomic and metabolomic are also applied to reveal the changes occurring under salt stress. By analyzing differential expressed genes (DEGs) under stress, transcription factors (TFs), such as myeloblastosis (MYB), WRKY and basic helix–loop–helix (bHLH), are demonstrated to play important roles in regulating gene expressions under salinity [68]. Responses of microalgae to both low-dose and high-dose salinity are shown in Table 3.

### 3.2. Salt Stress Strategies for Carotenoid Accumulation

Carotenoid production could be affected by environmental abiotic stress. Under salt stress, carotenoids will accumulate and protect cells as antioxidants to increase the surviving possibility of microalgae. In addition, optimal salt condition varies among different microalgal species. After NaCl treatment (1%, 0.17 M) for 10 days, the astaxanthin content of *H. pluvialis* climbed from 3.53 mg/g to 17.7 mg/g [87]. *C. zofingiensis* CCAP 211/14 can tolerate moderate NaCl concentration of 100 mM, and a significant enhancement of astaxanthin content was observed under 200 mM NaCl treatment [88]. The optimal condition to obtain the highest amount of fucoxanthin (79.40 ± 0.95 mg/g DW) of *Tisochrysis lutea* was determined as 36.27 g/L salt addition [64].

As the optimal conditions for cell growth are commonly different from those for secondary metabolite accumulation, two-stage cultivation has been widely applied to microalgae cultivation, a potent strategy to balance cell growth and metabolite accumulation. Acidophilic eukaryotic microalga *Coccomyxa onubensis* can endure moderate salt stress and serves as a potential resource for lutein production. Its growth rate and biomass productivity were significantly boosted under salt treatment (100 mM NaCl). By adding 500 mM NaCl, the lutein content was significantly enhanced by 47% to 7.80 mg/g DW, though the cell growth was inhibited [89]. Thus, cultures containing 100 mM salt can be applied at the first stage for higher biomass, then extra salt was added to induce lutein accumulation at the second stage.

Moreover, salt stress can also be coupled with other treatments to increase the carotenoid accumulation in microalgae. Light induction and nutrient starvation are widely used methods to boost carotenoid production. Combined with high light, salinity treatment increased the astaxanthin yield of *C. zofingiensis* 7.53-fold compared with the control [70]. *D. salina* was able to adapt to NaCl ranging from 0.05 to 5.5 M and the β-carotene content of *D. salina* achieved 13% of DW under salt stress, combined with high light at high temperature under nutrient deficiency [40].

The addition of chemicals can also boost carotenoid accumulation under salt stress. Photocatalyst TiO_2_ can enhance zeaxanthin accumulation under salt stress in *Coelastrella sp.* by increasing oxidative stress [42]. The maximum zeaxanthin (13.2 ± 4.4 mg/g DW) was achieved under high salinity (3% NaCl) and N-starvation at 40 °C after TiO_2_ treatment. An 0.25 mM amount of γ-aminobutyric acid (GABA) could facilitate astaxanthin productivity by 3.24-fold in *H. pluvialis* under high light with salinity treatment (2 g/L) [90]. Melatonin (MT) addition enhanced the expression of carotenogenic genes of *H. pluvialis* and induced astaxanthin accumulation by 1.20-fold under N-starvation and salt stress (1 g/L) [91]. NaCl treatment could also amplify the effect of linoleic acid (LA) on boosting astaxanthin accumulation in *Chlorella sorokiniana*, and LA could increase astaxanthin content by 1.25-fold in the presence of 20% NaCl (*w*/*v*) [92]. Strategies with salinity treatment to boost carotenoid accumulation in microalgae are summarized in Table 4.

In summary, moderate salt stress can increase biomass productivity and induce accumulation of carotenoids, depending on microalgae species, while strict stress will be toxic and even fatal to microalgae. As both high cell density and carotenoid productivity are desirable for biotechnological goals, an optimal salt concentration should be chosen cautiously according to different cultivation goals and microalgae species. Two-stage or multi-stage cultivation and strategies combining salinity with other stress factors also have potential in microalgae cultivation for carotenoids.

## 4. Potential Applications of Internet of Things (IoT) in Carotenoids Production

The Internet of Things (IoT) is a huge ecosystem that connects things, machines and humans, anytime and anywhere. As the third wave of the world information industry, the IoT has attracted thriving research interest from various fields, such as the food supply chain [93], healthcare applications [94] and precision agriculture [95]. The number of IoT devices worldwide is predicted to reach 75 billion by 2025 [93]. Considering its successful experiences in agriculture and its advantages as a real-time, efficient and intelligent comprehensive data system of works, the IoT also shows competitiveness in microalgae biorefinery [96].

**Figure 4:**
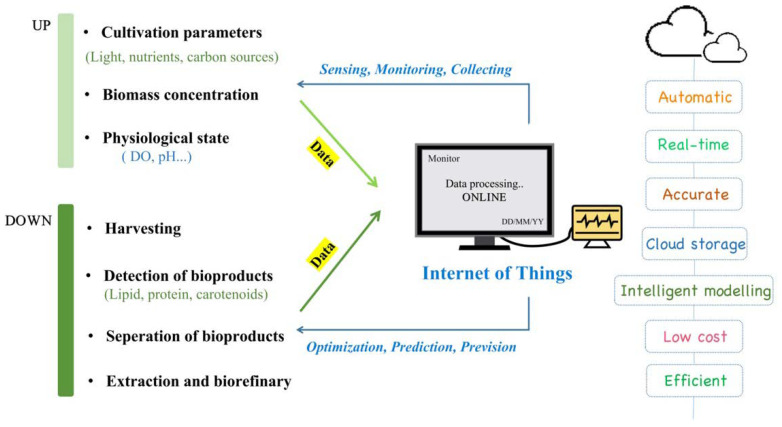
Characteristics of IoT and their potential utilizations in microalgae biorefinery. UP, upstream processing; DOWN, downstream processing.

Microalgae biorefinery can be divided into two consecutive categories, the upstream processing and downstream processing [97]. The upstream processing mainly focuses on the high-density cultivation of selected microalgae and the regulation and optimization of the cultivation process, such as choosing the suitable trophic mode and light conditions. The downstream process mainly includes the harvest of microalgae, the separation and extraction of key products and subsequent processing to make microalgae-derived products. The IoT, as an ubiquitous, huge network, can be perfectly combined with traditional biotechnologies in microalgae biorefinery [96]. As shown in Figure 4, both upstream and downstream events are connected and collected, and all information will then be processed by the IoT platform. Then, how can the IoT be applied in the production of microalgae-derived carotenoids?

For the upstream processing of carotenoid production, sensors are indispensable for data monitoring. Traditional detection techniques (such as measurement of biomass, cell size, etc.) are time-consuming, prone to causing culture contamination and cannot get data in time. In comparison, online sensors can monitor microalgae growth to gain real-time data, calculate unmeasured variables and even predict growth models.

For example, *H. pluvialis* is relatively sensitive to culture environment; its growth and cell composition can go far away from expectations without careful tuning. Possible contamination during sampling can disturb its growth. In addition, nutrient deficiency could drive it into a cyst structure and cause it to begin accumulating astaxanthin and other secondary metabolites [10]. In this case, innovative online sensors can monitor its growth in several aspects. Optical density (OD) sensors [98,99] can determine real-time biomass and dissolved oxygen (DO), CO_2_ sensors [100] can screen metabolic activity, pH sensors and nitrate and phosphorous sensors [101] can guarantee a suitable environment and nutrient sufficiency; these can all be applied to ensure high-density cultivation of *H. pluvialis*. Moreover, the IoT can also act as the base of a decision support system. By combining the data from sensors with simulation models, the IoT platform can predict growth trends and provide cultivation suggestions to maximize biomass production [102]. Afterwards, automation units of the IoT are essential to achieve condition debugging at the right time after data analysis, such as a suitable feeding strategy [103]. As for cultivation with salt-stress treatments, the IoT will facilitate picking the right timepoint to conduct salinity pressure to adequate microalgal cell density.

For the downstream processing of carotenoid production, it is particularly critical to distinguish microalgal cells from other contaminants in the culture medium. Since traditional turbidimeters detect both microalgal cells and pollutants, the measurement results are often not accurate enough [104]. As fluorescence only comes from chlorophyll pigment of alive cells, convenient chlorophyll fluorescence sensors have been designed and widely applied in in situ detection of microalgal cells with high efficiency and accuracy [105,106].

Take the astaxanthin production from *C. zofingiensis* as an example. *C. zofingiensis* can synthesize astaxanthin in adversity and store it in lipid droplets [55]. As it can take up glucose for rapid growth, it is more likely to be contaminated by bacteria during the actual cultivation process. Chlorophyll fluorescence sensors can visibly separate microalgae from bacteria and cell debris, which can be applied in quantification of microalgae. Additionally, bioproducts content can be non-invasively detected *in situ*, such as with a triacylglycerides (TAG) observer [107]. As for cultivation with salt-stress treatments, the IoT will facilitate in distinguishing cell debris under salt stress to accurately harvest and quantify microalgal cells, and a TAG observer can also display astaxanthin content to achieve preliminary evaluation of productivities. The IoT, as an intelligent system, can also choose the best extraction method after the target product has been separated, which saves time while ensuring purity and extraction efficiency.

## 5. Future Prospects

### 5.1. Genetic Modifications of Microalgae for Salt Tolerance and Carotenoid Accmulation

Microalgae are widespread and large in number, and only a small part of them have been studied. Therefore, there are still numerous novel microalgae species that may be able to tolerate salinity and accumulate carotenoids. Here, we mainly focus on three methods to get expected microalgae strains: (1) adaptive evolution, (2) random mutagenesis and (3) targeted genetic engineering.

Different to strain isolation, adaptive evolution can obtain an expected strain with a target phenotype. Adaptive evolution has been widely utilized for strain improvement to stress tolerance in various microalgae species, such as *C. reinhardtii* for 200 mM salt tolerance [108] and *Chlorella sp.* for high phenol concentration [109]. It was performed for improving the tolerance of a freshwater strain *Chlorella sp.* AE10 to 30 g/L salt for 138 days (46 cycles). The genes of the resulting strain *Chlorella sp.* S30 related to Calvin–Benson cycle, C4-dicarboxylic acid cycle and crassulacean acid metabolism (CAM) pathways were upregulated, which was beneficial for CO_2_ fixation under salt stress [85]. However, salinity acclimation of microalgae involves different functional genes and numerous pathways, which makes the exploitation of specific genes in salt tolerance more difficult.

Chemical mutagens, such as N-methyl-N’-nitro-N-nitrosoguanidine (MNNG) and ethyl methane sulfonate (EMS), are effective in mutant generation and may lead to mutants with enhanced carotenoid accumulation [110]. When genes in the carotenoid synthesis pathway (shown in Figure 1) are mutated, carotenoid profiles in microalgae may change. By chemical mutagenesis with EMS, carotenoid content in *Coelastrum sp.* C1-G1 was increased about 2-fold over its mother strain [39]. The Astaxanthin-overproduction strain *H. pluvialis* MT 2877 is a mutant by MNNG, which produced 4-fold astaxanthin over the WT strain [111]. *C. zofingiensis bkt1*, a chemical mutagen by MNNG, can accumulate high amounts of three essential carotenoids, e.g., zeaxanthin (7.00 ± 0.82 mg/g), lutein (13.81 ± 1.23 mg/g) and β-carotene (7.18 ± 0.72 mg/g) under different cultivation conditions [33], which can serve as a competent option for large-scale cultivation. *Chlorella sp.* was irradiated by ^137^Se- γ ray and domesticated with a seawater culture medium (salinity 3% wt.) under 15% CO_2_ stress. The biomass yield of the mutant was increased by 25% with 54.9% DW of lipids [112]. Although random mutagenesis could lead to novel traits, unpredictable traits may also show up, and the mutated genes still need further verification, such as gene sequencing, knockout and retro-complementation.

Targeted genetic engineering could generate specific insertions, deletions or substitutions in the host genome while avoiding random results [113]. Up until now, a lot of genes related to salt responses have been recognized, such as *nhaP* encoding a Na^+^/H^+^ antiporter, *codA* encoding choline oxidase to synthesize compatible solutes and *Dps* encoding DNA-binding proteins (Dps). Their introduction or overexpression strains have shown enhanced salt-tolerant abilities and potentials for seawater cultivation, which have been comprehensively reviewed [114].

Genetic engineering of microalgae for carotenoid hyperaccumulation is now a mature technology [115]. Genetic engineering of the green algae *C. zofingiensis* with a modified norflurazon-resistant endogenous *pds* gene resulted in up to 54.3% higher astaxanthin [116]. Transformation and expression of *dxs* and *psy* genes in *P. tricornutum* can increase fucoxanthin content by 2.4-fold and 1.8-fold, respectively [117]. *H. pluvialis* can also act as a gene donor. Transgenic *D. salina* with *bkt* and *chyb* genes from *H. pluvialis* was capable of astaxanthin biosynthesis with a better tolerance to high light [118].

At present, several microalgal genomes sequences are available in the public databases, such as *C. reinhardtii*, *Chlorella variabilis*, *Nanochloropsis graditana*, *C. zofingiensis*, etc. [119]. This offers worthy opportunities for precise engineering technology. Novel molecular tools have been applied to modify microalgal traits, including advanced genome editing systems, such as clustered regularly interspaced palindromic repeats and associated proteins (CRISPR-Cas), transcription activator-like effector nuclease (TALEN), zinc-finger nuclease (ZFN) and RNA interference (RNAi) [10,116]. For example, knocking down zeaxanthin epoxidase gene (*zep*) in C. *reinhardtii* by using DNA-free CRISPR-Cas9 RNP-mediated mutagenesis can increase zeaxanthin content by 56-fold [120].

### 5.2. Co-Production of Carotenoids with Value-Added Products from Microalgae

Lipids and several carotenoids (such as astaxanthin) are secondary metabolites of microalgae under adversity, which means certain stimulating conditions may promote the accumulation of two products simultaneously. In *H. pluvialis*, astaxanthin esterified by FAs is stored in lipid bodies, where TAGs are also positioned. Additionally, the biosynthesis of FAs and TAG has been demonstrated to be correlated with astaxanthin biosynthesis in *H. pluvialis* [121]. By adding 6 μM Cu^2+^, the astaxanthin and lipid content was increased by 66.87 and 34.99%, respectively, with carotenogenic and lipogenic genes being upregulated at transcriptional level [122].

Microalgae also contain biofunctional unsaturated fatty acids. Omega-3 polyunsaturated fatty acids (Omega-3 PUFA) from microalgae, such as eicosapentaenoic acid (EPA. C20:5) and docosahexaenoic acid (DHA, C22:6), have been proved to have various health effects with dietary intake, lowering the risks of neurodegenerative diseases and cardiovascular disorders [123]. *Nannochloropsis oceanica* oil is rich in EPA, while its zeaxanthin content (30.2 mg/g DW) is relatively high among microalgae species [41], making it a potential species for co-production of zeaxanthin and EPA. *O. aurita* also contains high concentrations of EPA (28% of total fatty acids), which has been confirmed for large-scale culture in open ponds [47], making it competent for producing both fucoxanthin (18.47 mg/g DW) and EPA.

In addition, co-production of fucoxanthin and DHA is feasible in microalgae. The marine algae genus *Isochrysis* is rich in DHA, and it allows easy extraction of DHA and fucoxanthin as it has no cell wall. Sun et al. screened 16 strains of *Isochrysis* for DHA and fucoxanthin production, and *Isochrysis* CCMP1324 in semi-continuous cultivation excels with productivities as 9.05 and 7.96 mg/L/day of DHA and fucoxanthin, respectively [124]. *I. zhanjiangensis* accumulated both high content of fucoxanthin (23.29 mg/g DW) and stearidonic acid (SDA, 17.16% of total fatty acid), which is the common precursor of EPA and DHA [46].

Additionally, both cultivation strategies and efficient extraction methods are worth noting. As zeaxanthin and lipid are soluble in organic solvents, choosing an efficient extraction system, such as hexane: methanol (3:2), can realize the best extraction efficacy for both EPA (36.4 mg/g DW) and zeaxanthin (28.2 mg/g DW), which also avoid the usage of toxic chloroform [41]. As most fucoxanthin was mostly in the hydroalcoholic phase (over 99%), lipid and fucoxanthin can be separated by this biphasic system after the same extraction process with pure ethanol solvent [50].

The residual biomass after carotenoid extraction of microalgae can be further processed to harvest other by-products. Under nutrient starvation, *D. salina* accumulated β-carotene and triglycerides up to 30–60% DW [125]. After carotenoid extraction, the residuals can be employed for co-production of biodiesel after transesterification. In addition, the carbohydrate in *D. salina* can also be employed for bioethanol production [126].

## 6. Conclusions

In this review, we comprehensively introduced health-promoting carotenoids in microalgae, summarized the carotenoid biosynthetic pathways and discussed microalgal responses to salt stress. Advances in applying the Internet of Things (IoT) in modern biotechnology and co-production of carotenoids with other high-value products were also considered in detail. Through in-depth research on the effects of salt stress on microalgae, it is believed that the cultivation of freshwater microalgae in seawater will no longer be an obstacle in the future. Moreover, it is also important to exploit more potential salt-tolerant microalgae species for high-yield production of biofunctional carotenoids.

## Figures and Tables

**Table 1:** Natural carotenoids and their natural sources, biofunctions and recommended doses.

Carotenoids	Natural Sources	Biofunctions	Recommended Dose	Ref.
**Lutein**	Marigold flower *; Yolk; Broccoli; Microalgae Orange-yellow fruits; Leafy green vegetables;	Antioxidant; Filter blue light; Prevent AMD; Prevent AD	6 mg day^−1^	[3,17]
**Astaxanthin**	Shrimp; Salmon; Crabs; Microalgae (Haematococcus pluvialis *) *Phaffia rhodozyma*	Antioxidant; Anti-aging; Anti-inflammatory; Anti-hypertensive; Anti-cancer;	4–12 mg day^−1^	[10,18]
**β-** **c** **arotene**	Pumpkin; Mango; Carrots; Microalgae (Dunaliella salina *)	Vitamin A precursor; Antioxidant; Anti-cancer; Anti-cardiovascular; Immune enhancement	600 μg RE ^1^/day	[19]
**Zeaxanthin**	Marigold flower *; Maize; Orange peppers; Microalgae; Scallions	Filter blue light; Improve visual acuity; Anti-cancer; Anti-inflammatory; Anti-allergy Against UV, skin redness	2 mg day^−1^	[13,20]
**Fucoxanthin**	Macroalgae *; Microalgae	Anti-cancer; Anti-hypertensive; Anti-inflammatory; Anti-obesity	−	[12,21]

Footnotes: DW, dry weight; AMD, age-related macular degeneration; AD, Alzheimer’s Disease. * This symbol represents the main source of a certain carotenoid. ^1^ RE, retinol equivalent.

**Table 2:** Microalgae-derived carotenoids, content and their biofunctions.

Carotenoid	Microalgae	Content	Productivity/Yield	Ref.
**Lutein**	*Chromochloris zofingiensis bkt1* (mutant)	13.81 mg/g DW	33.97 mg/L	[33]
	*Parachlorellasp.* JD-076	11.87 mg/g DW	25.0 mg/L/day	[32]
	*Chlorella sorokiniana* FZU60	11.22 mg/g DW	8.25 mg/L/day	[29]
	*Chlorella vulgaris* UTEX 265	9.82 mg/g DW	11.98 mg/g/day	[34]
	*Chlorella vulgaris* CS-41	9.0 mg/g DW	1.56 mg/L/day	[35]
	*Scenedesmus* sp.	7.47 mg/g DW	19.70 mg/L/day	[36]
	*Chlorella sp.* GY-H4	8.9 mg/g DW	10.50 mg/L/day	[28]
	*Chlorella sorokiniana* MB-1-M12	7.39 mg/g DW	3.43 mg/L/day	[27]
	*Chlorella minutissima* MCC-27	7.05 mg/g DW	6.34 mg/L/day	[26]
**Astaxanthin**	*Haematococcus pluvialis*	5% DW	65.8 mg/m^2^/day	[37]
	*Chromochloris zofingiensis*	6.5 mg/g DW	0.8 mg/L/day	[38]
	*Coelastrum sp.* G1-C1 (mutant)	−	28.32 mg/L	[39]
**β-** **c** **arotene**	*Dunaliellasalina* *	13% DW	−	[40]
	*Chromochloris zofingiensis bkt1* (mutant)	7.18 mg/g DW	34.64 ± 1.39 mg/L	[33]
**Zeaxanthin**	*Nannochloropsis oceanica* CCNM 1081 *	30.2 mg/g DW	−	[41]
	*Coelastrella sp.* M60	13.15 mg/g DW	0.72 mg/L/day	[42]
	*Chromochloris zofingiensis bkt1* (mutant)	7.00 mg/g DW	36.79 ± 2.23 mg/L	[33]
	*Dunaliellasalina zea1* (mutant) *	5.9 mg/g DW	−	[43]
	*Chlorella ellipsoidea* *	4.26 mg/g DW	−	[44]
**Fucoxanthin**	*Mallomonas sp.*	26.6 mg/g DW	−	[45]
	Isochrysis zhanjiangensis *	23.29 mg/g DW	2.94 mg/L/day	[46]
	Odontella aurita *	18.47 mg/g DW	7.96 mg/L/day	[47]
	*Isochrysis* aff. Galbana *	18.23 mg/g DW	−	[48]
	Tisochrysis lutea *	16.39 mg/g DW	9.81 mg/L/day	[49]
	Phaeodactylum tricornutum *	16.33 mg/g DW	−	[50]

* indicates seawater microalgae species.

**Table 3:** Overview of the effects of salinity on microalgae at different doses.

Responses	Low-Dose NaCl	High-Dose NaCl
Physiology	Growth ↑; Photosynthesis ↑	Growth ↓; Chlorophyll content↓; Photosynthesis ↓
Morphology	No significant changes	Cell size ↑; Cell wall ↑ Color change
Main carbon sinks	Carbohydrate ↑	Carbohydrate (providing building blocks) ↓ TAGs ↑
Gene expression	*nr* gene (for N assimilation) ↑ Photosynthetic enzyme genes (*rbcL*, *rbcS*) ↑	TFs (WRKY, MYB, bHLH...) Antioxidant enzymes genes (SOD, CAT) ↑ Secondary metabolites genes ↑ Fatty acid synthesis genes ↑ Starch catabolism genes (PK) ↑ Gluconeogenesis gene (PEPCK) ↓
Metabolites	Lutein ↑	Secondary carotenoids ↑ TFA ↑

**Table 4:** Salt treatment and salinity-combined conditions for carotenoids production.

Stress Conditions	Microalgae	Carotenoids	Fold Change	Ref.
100–500 mM NaCl (Two-stage)	*Coccomyxa onubensis*	Lutein	0.47-fold	[89]
200 mM NaCl	*C. zofingiensis* CCAP 211/14	Astaxanthin	1.23-fold	[88]
36.27 g/L NaCl	*Tisochrysis lutea*	Fucoxanthin	−	[64]
2% NaCl (*w*/*v*)	*Chromochloris zofingiensis bkt1*	Zeaxanthin	1.38-fold	[33]
		Lutein β-carotene	0.22-fold	
			0.36-fold	
High light + NaCl	*Chromochloris zofingiensis*	Astaxanthin	7.53-fold	[70]
LA + NaCl (20%)	*Chlorella sorokiniana*	Astaxanthin	1.25-fold	[92]
GABA + high light + NaCl	*Haematococcus pluvialis*	Astaxanthin	3.24-fold	[90]
MT+ N-starvation + NaCl	*Haematococcus pluvialis*	Astaxanthin	1.20-fold	[91]
TiO_2_ + N-starvation + NaCl	*Coelastrella sp.*	Zeaxanthin	0.51-fold	[42]
		Astaxanthin	1.16-fold	

## Data Availability

Not applicable.
